# Risk-Prediction Models for Clinical Decision-Making in Sarcoma Care: An International Survey Among Soft-Tissue Sarcoma Clinicians

**DOI:** 10.1245/s10434-024-16849-7

**Published:** 2025-02-01

**Authors:** Anouk A. Kruiswijk, Lisa A. E. Vlug, Ibtissam Acem, Ellen G. Engelhardt, Alessandro Gronchi, Dario Callegaro, Rick L. Haas, Robert J. P. van de Wal, Michiel A. J. van de Sande, Leti van Bodegom-Vos

**Affiliations:** 1 Department of Biomedical Data Sciences, Medical Decision Making, Leiden University Medical Center, Leiden, The Netherlands; 2 Orthopedic Surgery, Leiden University Medical Center, Leiden, The Netherlands; 3 Department of Surgical Oncology and Gastrointestinal Surgery, Erasmus MC Cancer Institute, Erasmus Medical Center, Rotterdam, the Netherlands; 4 Division of Psychosocial Research and Epidemiology, The Netherlands Cancer Institute, Amsterdam, The Netherlands; 5 Department of Surgery, Fondazione IRCCS Istituto Nazionale dei Tumori, Milan, Italy; 6 Department of Radiotherapy, The Netherlands Cancer Institute, Amsterdam, the Netherlands; 7 Department of Radiotherapy, Leiden University Medical Center, Leiden, the Netherlands

## Abstract

**Introduction:**

Risk prediction models (RPMs) are statistical tools that predict outcomes on the basis of clinical characteristics and can thereby support (shared) decision-making. With the shift toward personalized medicine, the number of RPMs has increased exponentially, including in multimodal sarcoma care. However, their integration into routine soft-tissue sarcoma (STS) care remains largely unknown. Therefore, we inventoried RPM use in sarcoma care during tumor board discussions and patient consultations as well as the attitudes toward the use of RPMs to support (shared) decision-making among STS clinicians.

**Materials and Methods:**

A 29-item survey was disseminated online to members of international sarcoma societies.

**Results:**

This study enrolled 278 respondents. Respectively, 68% and 65% of the clinicians reported using RPMs during tumor board discussions and/or patient consultations. During tumor board discussions, RPMs were used primarily to assess the potential benefits of (neo)adjuvant chemotherapy. During patient consultations, RPMs were used to predict patient prognosis upon request and to assist in decision-making regarding (neo)adjuvant therapies. The reliability of patient risk predicted by RPMs and the absence of guidelines regarding the use of RPMs were identified as barriers. Additionally, some clinicians questioned the applicability of estimates from RPMs to individual patients and expressed concerns about causing unnecessary anxiety when discussing prognostic outcomes.

**Conclusions:**

Responding STS clinicians frequently use RPMs to support decision-making about (neo)adjuvant therapies. However, they expressed concerns about the applicability of RPM estimates to individual patients and reported challenges in communicating prognostic outcomes with patients. These findings highlight the difficulties clinicians face when integrating RPMs into patient consultations.

In recent years, risk prediction models (RPMs) have emerged as valuable tools that can support clinicians in practicing evidence-based medicine. RPMs are statistical tools that can predict the likelihood of a certain outcome for an individual on the basis of their (clinical) characteristics.^1^ By considering multiple parameters simultaneously, RPMs have the potential to provide a more comprehensive and accurate assessment of individual patient risk than to traditional approaches do. Furthermore, RPMs can also support (shared) decision-making during clinical encounters. Given that patients’ goals and preferences for treatment vary widely and that RPMs allow personalized risk assessment, the information from RPMs provide may empower patients to weigh their options and engage more actively in the decision-making process.^2,3^

The number of RPMs to support adequate management of diseases has increased exponentially over the last decade, given the move toward more personalized medicine, specifically in oncology and multimodal soft-tissue sarcoma care.^4,5^ Soft-tissue sarcomas (STSs) are rare and heterogeneous malignant neoplasms, with more than 100 histological subtypes.^6^ They arise from mesenchymal cells and account for 1% of all adult malignancies, with an estimated incidence of approximately 5 cases per 100,000 individuals in Europe per year.^7,8^ STSs can occur at any anatomical site, but they predominantly manifest in the extremities.^8^ Because of the heterogeneity in presentation and outcome, clinicians experience distinct difficulties in diagnosis, treatment, and follow-up of STSs.

Acem et al. provided a clear overview of published RPMs for patients with primary STSs.^4^ Two prediction tools for patients with soft-tissue sarcoma of the extremities (eSTS), Sarculator^9^ and PERsonalised SARcoma Care (PERSARC),^10,11^ have undergone external validation^12–16^ and provide dynamic predictions, allowing for adjustments in the predicted prognosis as the patient’s condition improves over time. Both tools can be accessed on a smartphone, facilitating their use in clinical practice. Despite potential utility, the extent to which RPMs, such as Sarculator and PERSARC, have been integrated into routine clinical decision-making for sarcoma care is largely unknown. The aim of this study is to address this gap by examining the current utilization of RPMs in sarcoma care. Specifically, we seek to assess the extent and reasons for RPM use or nonuse by clinicians, both within the context of multidisciplinary tumor boards and during patient consultations. Additionally, given the potential of RPMs to support shared decision-making, we aim to evaluate the general attitudes toward shared decision-making regarding STS treatment among clinicians and the role of RPMs in supporting this process in sarcoma care.

## Materials and Methods

### Survey Design and Measures

The survey used in this study was developed after a literature review by the project team, which consisted of an expert in risk-prediction models, an implementation scientist, and three orthopedic/oncological surgeons. The survey was internally tested for content and validity by the study team and two orthopedic surgeons at the Leiden University Medical Center in the Netherlands. It was created and distributed using online survey software (Castor EDC, 2019)^17^ and was available to respondents from 16 January to 1 May 2024.

The survey included questions regarding respondent characteristics (age, sex, specialty, location of practice, years of experience, and number of new cases diagnosed per year), extent to which RPMs are currently used in tumor board discussions and in clinical consultations with primary eSTS patients [5-point Likert scale (never–always)] and reasons for using or not using RPMs during tumor board discussions and clinical consultations (multiple choice). Additionally, clinicians were asked to rate the percentage of patients with eSTS for whom more than one reasonable treatment option existed (0–100%) to acknowledge how frequently they discussed reasonable treatment options with these patients with eSTS, with the aim of engaging in shared decision-making regarding personalized treatment plans (never–always). In eSTS, the uncertainty around treatment outcomes, along with the tradeoff between risks and benefits and patient preferences, might result in a greater number of reasonable treatment options. Clinicians’ willingness to incorporate shared decision-making into clinical practice for patients with eSTS was measured using the median score of items 1–5 from the IncorpoRATE tool (resulting in a score between 0% and 100%, with higher scores indicating greater willingness to adopt shared decision-making).^18^ As this survey was intended to be completed by clinicians, we used mainly closed-ended questions and allowed the respondents to skip questions to ensure that they completed the survey within 10 min. The 29-item survey is available in Appendix 1.

### Study Population

The target population for this survey study consisted of clinicians (i.e., medical oncologists, orthopedic oncologists, radiation oncologists, and surgical oncologists) who were responsible for treating patients with eSTS. The study population was invited to complete the survey via an email that was sent by the European Musculo-Skeletal Oncology Society (EMSOS), the Musculoskeletal Tumor Society (MSTS), the Connective Tissue Oncology Society (CTOS), and the Asia Pacific Musculoskeletal Tumor Society (APMSTS) to their members. This email detailed the survey’s objectives and included a link to access the online survey. A reminder was sent after 1 month. To minimize the risk of duplicate responses of one participant, we included a clear note in both the invitation and reminder emails, emphasizing the anonymous nature of the survey distribution through various sarcoma societies and explicitly requesting respondents not to complete the survey more than once.

### Statistical Analysis

Data from all respondents who indicated that they treated patients with eSTS and who had completed at least the background characteristics section and one other survey question were included in the analyses. Descriptive statistics were used to describe the background characteristics, RPM use, and perspectives on shared decision-making. The findings for each survey question about RPM use and perspectives on shared decision-making are reported as percentages, along with the number of relevant respondents and the total *N* of respondents who answered the question [e.g., % (*n*/*N*)], as the number of respondents per question varies owing to incomplete survey responses and conditional questions.

The main questions regarding RPM use in tumor board discussions and clinical consultations, and the attitudes toward (shared) decision-making were stratified by specialty and continent. The respondents from Central and South America, Africa, Australia, New Zealand, and Oceania were excluded from the analysis stratified by continent owing to small sample sizes (*n* < 40). All analyses were performed using the statistical program SPSS (IBM SPSS, version 29).

The reporting of this research is in accordance with the Consensus-Based Checklist for Reporting of Survey Studies (CROSS).^19^ The study was approved by the Medical Ethical Committee Leiden-Den Haag-Delft (2024-022) and adhered to regulations governing Good Clinical Practice and General Data Protection Regulation.

## Results

In total, 278 of the 324 (86%) respondents who started the survey met the inclusion criteria. Respondents were excluded for the following reasons: not being specialized in treating eSTS (*n* = 17), not completing the section on baseline characteristics (*n* = 26), and/or not answering any survey question beyond the background characteristics (*n* = 3). In total, 240 of the 278 (86%) respondents included in the analyses completed the entire survey. The response rate could not be calculated because the survey respondents were anonymous, and eligible clinicians may have belonged to multiple societies.

Appendix 2 contains a flowchart detailing the respondent inclusion criteria.

Most respondents were orthopedic oncologists (46%, *n*/*N* = 127/278) who practiced in Europe (51%, *n*/*N* = 145/278) and had more than 10 years of experience treating patients with eSTS (54%, *n*/*N* = 150/278) (Table 1).

**Table 1:** Baseline characteristics of the respondents

Characteristics	Overall *N* = 278
Age, years (median, IQR)	45 (40–52)
Sex (*n*, %) Male	185 (66.5)
Specialty (*n*, %)	
Medical oncology	79 (28.4)
Orthopedic oncology	127 (45.7)
Radiation oncology	25 (9.0)
Surgical oncology	40 (14.4)
Other*	7 (2.5)
Years of experience in treating eSTS patients (*n*, %)	
< 1 year	7 (2.5)
1–2 years	11 (4.0)
3–5 years	47 (16.9)
6–10 years	63 (22.7)
> 10 years	159 (54.0)
Current practice location (*n*, %)	
Africa	4 (1.4)
Asia	39 (14.0)
Australia/New Zealand/Oceania	13 (4.7)
Central/South America	3 (1.1)
Europe	143 (51.2)
North America	76 (27.3)
Number of new cases diagnosed annually (*n*, %)	
< 5 per year	3 (1.1)
5–25 per year	53 (19.1)
25–50 per year	67 (24.1)
50–75 per year	155 (55.8)

^*^Including pediatric and adolescent oncology and pathology

*IQR* interquartile range, *eSTS* soft-tissue sarcoma of the extremities

### Use of RPMs in Multidisciplinary Tumor Boards

**Fig. 1:**
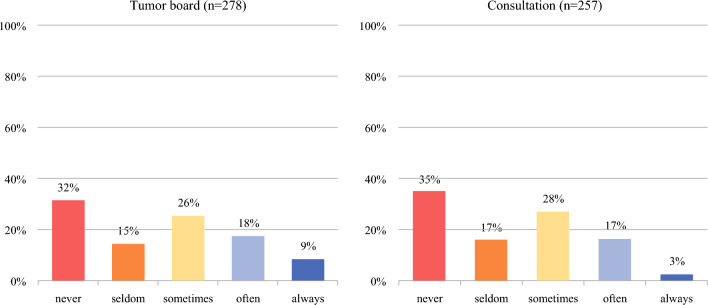
Current use of RPMs in multidisciplinary tumor boards and during patient consultations

Among 278 respondents, 68% (*n*/*N* = 190/278) reported using an RPM in multidisciplinary tumor boards, 27% (*n*/*N* = 76/278) of whom reported frequent use (often/always) (Fig. 1). In addition, 32% (*n*/*N* = 88/278) indicated that they never used an RPM during tumor board discussions. The assessment of RPM use during tumor board discussions stratified by clinical specialty revealed that medical oncologists (44%, *n*/*N* = 35/76) utilized an RPM (Appendix 3) more often than other types of STS clinicians. Descriptive analysis of RPM use among multidisciplinary tumor boards by geographic location revealed that clinicians from Europe more frequently used RPMs often/always (38%, *n*/*N* = 54/143) than clinicians from North America (22%, *n*/*N* = 17/76) or Asia (3%, *n*/*N* = 1/39). Sarculator and PERSARC were the two most frequently used RPMs either individually or in combination; among respondents who reported using an RPM in multidisciplinary tumor boards, 87% (*n*/*N* = 163/1878) reported that they use Sarculator for an average of 31% (interquartile range, IQR 15–70%) of their patients, 33% of respondents (*n*/*N* = 62/187) indicated using PERSARC for, on average, 25% (IQR 10–70%) of the patients discussed, and 23% of the respondents (*n*/*N* = 43/187) indicated using Sarculator and PERSARC.

The main reasons reported for RPM use during tumor board discussions were the following: (1) the added value of (neo)adjuvant chemotherapy (CTx) (70%, *n*/*N* = 126/181), (2) the impact of therapy on prognosis (57%, *n*/*N* = 103/181), or (3) the impact of the combination of (neo)adjuvant CTx and radiotherapy (RTx) on prognosis (23%, *n*/*N* = 42/181). However, clinicians indicated that their personal preferences for treatment modalities (RTx/CTx) are as important as the RPM results regarding overall survival (OS) and local control (LR) when determining the added value of offering a patient recommending RTx or CTx (58%, *n*/*N* = 103/177). Nonusers identified several barriers to using RPMs in tumor board discussions, including (1) perceived inaccuracy of the risk estimates provided by RPMs (40%, *n*/*N* = 21/52) and (2) the lack of guidelines on the incorporation of RPMs (40%, *n*/*N* = 21/52).

### Use of the RPM during Consultation with the Patient

Of the 257 clinicians who answered the question, 65% (*n*/*N* = 166/257) reported using a RPM during consultations with the patients, whereas 35% (*n*/*N* = 91/257) never used it during consultations. Compared with their counterparts in North America (17%, *n*/*N* = 12/69) and Asia (3%, *n*/*N* = 1/36), clinicians in Europe most frequently reported using RPMs often or always during consultation (26%, *n*/*N* = 35/133). Among clinicians experienced in STS care, medical oncologists were the most likely to use RPMs during patient consultation, with 39% (*n*/*N* = 27/69) doing so regularly (see Appendix 3). Sarculator was the most used RPM (85%, *n*/*N* = 139/164) among the respondents who reported using an RPM during consultations, and was employed in approximately 30% (IQR 15–68%) of patient consultations. PERSARC was the second most frequently used tool (33%, *n*/*N* = 54/164) and was utilized in 24% (IQR 12–70%) of patient consultations. A total of 20% (*n*/*N* = 33/164) reported using both Sarculator and PERSARC, whereas 4% (*n*/*N* = 6/164) reported using another model (for example, the MSKCC nomograms).

The most cited reasons for using a RPM during patient consultations were: (1) to provide prognostic estimates when requested by patients (65%, *n*/*N* = 104/161) and (2) to assist patients in making decisions about (neo)adjuvant CTx (57%, *n*/*N* = 91/161). Conversely, the primary reasons for not using an RPM during consultations, cited by nonusers, were as follows: (1) the perception that RPMs do not accurately reflect the specific risks for individual patients, as clinicians feel that the risks apply to groups (54%, *n*/*N* = 29/54), and (2) the belief that disclosing personalized risks to patients causes unnecessary anxiety (43%, *n*/*N* = 23/54).

### Perspective on Shared Decision-Making in eSTS

**Fig. 2:**
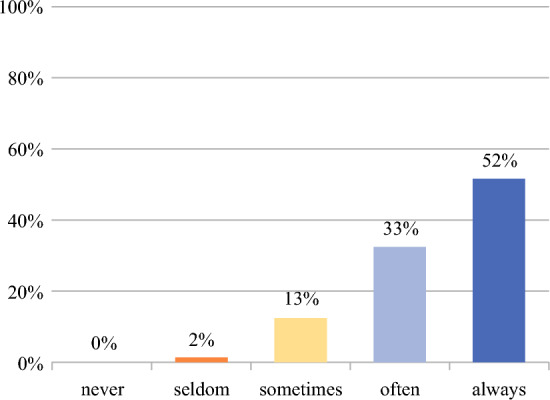
Extent to which treatment options are discussed with patients to collaboratively decide on personalized treatment plan (*n* = 243)

Clinicians (*n* = 247) reported that for 50% (IQR 30–75%) of the patients with STS who they treat, more than one reasonable treatment option exists; there was no substantial differences in clinicians’ perspectives between continents or specialties (Appendix 3). Clinicians indicated that they almost always discuss these reasonable treatment options with their patients (in 85% of cases) to include the patient in personalizing their treatment plan (Fig. 2). The involvement of patients in decision-making (73%, *n*/*N* = 174/240) and the discussion of the risks and benefits of different treatment options with patients (7%, *n*/*N* = 177/240) are the main arguments for clinicians to use an RPM to support (shared) decision-making (Fig. 2).

The respondents were willing to engage with patients with eSTS in (shared) decision-making; the overall median score on the IncorpoRATE scale was 83.8 out of 100 (Table 2).

**Table 2:** Median overall and per item incorpoRATE scores (n = 240)

Item	Description	Median	IQR	Range
1	Necessity in practice “In my opinion, when more than one reasonable option exists, educating patients about their treatment options eliciting their preferences, and coming to a collaborative decision is (unnecessary, 0—necessary, 100)”	95.0	79–100	8–100
2	Patient desirability “In my opinion, when more than one reasonable option exists, educating patients about their treatment options, eliciting their preferences, and coming to a collaborative decision is (not welcomed by patients, 0—welcomed by most patients, 100)”	78.0	52–95	4–100
3	Effective resource use “In my opinion, when more than one reasonable option exists, educating patients about their treatment options, eliciting their preferences, and coming to a collaborative decision is (poor use of my time,0—good use of my time, 100)”	86.0	70–100	6–100
4	Confidence in skill “In my opinion, when more than one reasonable option exists, educating patients about their treatment options, eliciting their preferences, and coming to a collaborative decision is (a skill I do not feel confident in, 0—a skill I do feel confident in, 100)”	80.0	60–100	1–100
5	Importance SDM despite clinical recommendations “In my opinion, when more than one reasonable option exists, educating patients about their treatment options, eliciting their preferences, and coming to a collaborative decision is (not important if there is a strong clinical preference, 0—important even if there is a strong clinical preference, 100)”	80.0	50–100	1–100
	Median incorpoRATE score	83.8	62–99	

## Discussion

The present study explored the utilization patterns and perceptions of RPMs among STS clinicians during tumor board discussions and patient consultations and focused on their potential for supporting (shared) decision-making in patients with eSTS. Almost two-thirds of the respondents indicated that they use an RPM during tumor board discussions and/or patient consultations, and approximately one-quarter indicated routinely using RPMs during tumor board discussions and/or patient consultations. Sarculator emerged as the most commonly used RPM, particularly during tumor board discussions and 30% of patient consultations on average. The main reason respondents cited using RPMs during tumor board discussions was to gain insight into the added value of (neo)adjuvant CTx, whereas during consultations, RPMs served to provide prognostic estimates upon patient request and to facilitate decision-making regarding (neo)adjuvant therapies. The reliability of prognostic estimates and a lack of guidelines regarding the incorporation of RPMs were perceived barriers. Moreover, some clinicians have expressed concerns regarding making patients unnecessarily anxious when providing prognostic estimates. Clinicians were highly motivated to engage in shared decision-making (SDM) and indicated that, generally, approximately half of their patients have a choice between multiple clinically viable options.

### Use of RPMs in Sarcoma Care

This study revealed that only a few RPMs, specifically Sarculator and PERSARC, have been integrated into the clinical care of patients with eSTS, and are used during both tumor board discussions and patient consultations. Insights into the added value of (neo)adjuvant therapies was the most frequently mentioned reason for using RPMs, which is consistent with previous studies on RPM use by both breast cancer specialists^20,21^ and sarcoma specialists.^22^ Our respondents, many of whom were medical oncologists, reported the use of RPMs to identify high-risk patients who may benefit from CTx, which aligns with the growing evidence supporting this approach.^9,23,24^ The European Society for Medical Oncology (ESMO) has recently recommended the use of RPMs in identifying high-risk patients for systemic therapies in its guidelines,^25^ and it is expected that other international societies will follow suit. However, there are also significant barriers to RPM use, particularly during patient consultations. These barriers include clinicians’ concerns that presenting these risks may cause unnecessary anxiety and the applicability of the RPM for selected patients. Consequently, some clinicians experienced in treating STS only share prognostic estimates if requested by patients, suggesting that clinicians may experience difficulties in communicating these estimates.^20,26^ Additionally, clinicians’ difficulties in fully understanding and effectively communicating these estimates to patients may further limit the use of RPMs in supporting shared decision-making.^27,28^ Future research should focus on the integration of RPMs into patient consultations to better understand their impact on the patient–clinician decision-making process.

### Perspectives on Shared Decision-Making among Clinicians Experienced in Treating eSTS

Responding clinicians reported that approximately half of the patients with eSTS who they treat have multiple clinically viable options from which they can choose, necessitating shared treatment decision-making.^29^ Often, clinicians unilaterally make subjective tradeoffs between the advantages and disadvantages of treatment options prior to consultations in multidisciplinary tumor boards.^30^ This raises questions about whether their evaluations align with those of their patients. To capitalize on clinicians’ willingness to adopt shared decision-making, it is essential to raise their awareness that maximizing disease-free survival is not always the primary treatment goal for all patients.^31^ Therefore, making these decisions collaboratively with the patient is crucial.^32^ Except in cases where it is medically inadvisable to provide treatment (e.g., patients with very low risk) or impossible to withhold treatment (e.g., patients with very high risk), there is often a choice, even if selecting one option does not lead to maximum survival.y^29^ International guidelines can play a significant role in this context. Reaching a consensus on the minimum survival benefit cutoff to at least offer treatment can better support the implementation of shared decision-making, aided by RPMs.

### Limitations

This study has some limitations. First, the anonymous electronic distribution of the survey, while enabling convenient delivery and response, prevented us from accurately determining the response rate. Second, selection bias is an inherent limitation of survey use, as clinicians who are interested in the topic are more likely to participate than those who are less interested in the topic. This may have led to an overestimation of the actual use of RPMs in clinical practice and desirability or reporting bias regarding shared decision-making. Additionally, since the survey was exclusively sent to active members of selected sarcoma societies, certain continents and specialties (e.g., radiation oncologists) were underrepresented, which may limit the generalizability of our findings. Nonetheless, the high completion rate (86%) indicates that the participating clinicians perceived the questions as sufficiently important to warrant their participation in the survey. At last, the term “risk prediction model” was not explicitly defined in the survey. This may have led to variability in how respondents interpreted it, and consequently may have influenced our results to a limited extent.

## Conclusions

Responding clinicians reported that RPMs are used in sarcoma care primarily to identify high-risk patients for (neo)adjuvant CTx, provide patients with prognostic estimates, and facilitate decision-making regarding (neo)adjuvant therapies. However, clinicians expressed concerns about the applicability of RPMs to individual patients and the potential negative impact, such as inducing anxiety, that this information can have on patients. These concerns highlight barriers that clinicians perceive in the use of RPMs, particularly during patient consultations. While clinicians are willing to adopt shared decision-making in sarcoma care, they need support to better integrate the use of RPMs in shared treatment decision-making in clinical practice.

## Data Availability

The data that support the findings of this study are available from the corresponding author, upon reasonable request.

## Appendix 1 Survey orthopedic/oncological sarcoma surgeon

“*What is this survey about?*

The number of risk prediction models (RPMs) to support adequate management of diseases has increased exponentially over the last decade in medicine, specifically in oncology and also in the field of multimodality soft-tissue sarcoma care. With this survey we aim to assess the use of and the barriers and facilitators for the use of RPMs by physicians for clinical decision-making in sarcoma care. Your input is highly appreciated to optimize the implementation of RPMs in sarcoma care to the needs of professionals involved in the treatment of patients with soft tissue sarcomas.

The survey consists of 29 questions and takes about 10 minutes to complete.

This research is conducted by the Leiden University Medical Center and The National Cancer Institute of Milan. The survey is completely anonymous, and the results cannot be traced back to individuals. By filling in the survey, you grant us permission to use the data for this study.

## Thank you for dedicating your time to fill in this survey.

Respondent characteristics

1. Do you treat extremity soft-tissue sarcoma(s) (eSTS) patients?
  - Yes
  - No -> end of survey
2. How many new cases of eSTS do you treat in your hospital annually?
  - < 5 per year
  - 5–25 per year
  - 25–50 per year
  - 50–75 per year
  - ≥ 75 per year
3. What is your specialty?
  - Medical oncology
  - Orthopedic oncology
  - Radiation oncology
  - Surgical oncology
  - Other
4. How many years of experience do you have in treating eSTS patients?
  - < 1 year
  - 1–2 years
  - 3–5 years
  - 6–10 years
  - > 10 years
5. Where do you practice?
  - Africa
  - Asia
  - Australia/ New Zealand /Oceania
  - Central/ South America
  - Europe
  - North America
6. What is your age? (Open)
7. What is your gender?
  - Male
  - Female
  - Other
  - Prefer not to say

Risk prediction models

**Risk prediction models** (**RPM**) are statistical tools which can predict the likelihood of a certain outcome or event for an individual based on their (clinical) characteristics. With the following questions we want to ascertain which **RPM** you are familiar with, which you may use and what you think of them.

8. Do you currently use a **RPM** for eSTS patients in **multidisciplinary tumor boards** (**MTB**)?

9. Are you familiar with **RPM** in eSTS patients, but currently don’t use them in **multidisciplinary tumor boards** (MTB)?
  - Yes = 16 question 16
  - No = 18 question 18
10. Which of the following **RPM** do u currently use in **MTB**? (multiple answer possible)
  - Persarc
  - Sarculator
  - Other, …
11. In which percentage of eSTS patients who are discussed during **MTB** do you use Persarc/Sarculator/other?
  - slider 0–100 %
12. Why do you use a RPM in multidisciplinary tumor boards? To get insight in the added value of: (multiple answers possible)

- □ (neo)adjuvant radiotherapy
- □ (neo)adjuvant chemotherapy
- □ the combination of chemotherapy and radiotherapy
- □ the impact of therapy on prognosis
- □ other, namely.. -> 13

13. Please indicate other
14. Which of the statements about the use of RPMs is most applicable to you?

- □ My personal preference for treatment modalities (CT/RT) weighs more in the decision to give RT and/ or CT than the results of the prediction model in terms of overall survival and local control -> 15
- □ The results of the prediction model in terms of overall survival and local control weigh more in the decision to give RT and/ or CT than my personal preferences for treatment modalities. -> 18
- □ My personal preferences and results of the prediction model in terms of overall survival and local control are equally important in the decision to give a patient RT and/ or CT. -> 18

15. What are your reasons for deviating from the RPM?

- □ open field -> 18

16. You have indicated that you currently do not use an risk-prediction model during MTB. Below are a few arguments to use RPMs in MTB in the future. Please indicate for each statement whether it applies to you. (multiple answers possible)

I would consider using an RPM in MTB in the future to get insight in the added prognostic value of:

- □ (neo)adjuvant radiotherapy
- □ (neo)adjuvant chemotherapy
- □ the combination of chemotherapy and radiotherapy
- □ the impact of therapy on prognosis
- □ other, namely…

17. Below are a few arguments against the use of a risk-prediction models in MTB that are sometimes made by clinicians. Please select the statement(s) to which you are in agreement? (multiple answers possible)

Information from RPM in MTB:

- □ does not add value to my expertise
- □ is not sufficiently scientifically supported – accuracy of estimates are not reliable
- □ is not sufficiently scientifically supported – important predictor variables are missing
- □ does not say anything about individual patients, as it applies to groups
- □ does not have a user-friendly interface
- □ is not incorporated in the electronic medical record
- □ is not incorporated in guidelines
- □ other ….

18. Do you currently use a **RPM** in **clinical consultation** with the eSTS patient?

19. Are you familiar with RPM in eSTS patients, but currently don’t use them in clinical consultation with the patient?
  - Yes = 23
  - No = 25
20. Which of the following RPM do u currently use in consultation with the eSTS patient? (multiple answer possible)
  - Sarculator
  - Persarc
  - Other...
21. In which percentage of eSTS patients do you use Persarc/Sarculator/other during clinical consultation?

- Slide 0–100%

22. Why do you use an RPM in clinical consultations? Please select the statement(s) to which you are in agreement. (multiple answers possible) -> question 24

I currently use RPM during clinical consultations:

- □ to inform patients
- □ to inform patients *if* they ask for prognostic estimates
- □ to convince patients about the usefulness of the treatment plan I am proposing
- □ to help patients to make a decision about (neo)adjuvant RTx
- □ to help patients to make a decision about (neo)adjuvant CTx
- □ to also present the chances graphically
- □ other…

23. You have indicated that you currently do not use an **RPM** during **clinical consultations**. Below are a few arguments to use RPM in clinical consultation in the future. Please select the statement(s) to which you are in agreement? (multiple answers possible)

I would consider using an RPM in clinical consultation in the future:

- □ to inform patients
- □ to inform patients *if* they ask for prognostic estimates
- □ to convince patients about the usefulness of the treatment plan I am proposing
- □ to help patients to make a decision about (neo)adjuvant RTx
- □ to help patients to make a decision about (neo)adjuvant CTx
- □ to also present the chances graphically
- □ other…

24. Below are a few arguments against the use of an RPM in clinical consultations that are sometimes made by clinicians. Please select the statement(s) to which you are in agreement? (multiple answers possible)

Information from risk prediction models in clinical consultation:

- □ is not sufficiently scientifically supported—accuracy of estimates are not reliable
- □ is not sufficiently scientifically supported—important predictor variables are missing
- □ does not say anything about individual patients, as it applies to groups
- □ is too complicated for patients
- □ makes patients unnecessarily anxious
- □ it takes up too much time in consultation
- □ does not have a user friendly interface
- □ is not incorporated in the electronical medical record
- □ is not incorporated in the guidelines
- □ other…

25. Would you consider using a **RPM** to individualize **follow-up** strategies?

- □ yes
- □ no
- □ maybe
- □ I already use a (dynamic) RPM to individualize follow-up strategies

SDM

Shared decision-making is a collaborative process in which healthcare professionals and patients work together to make decisions about the patient’s healthcare. It involves discussing reasonable treatment options, weighing the potential benefits and risks, and considering patient’s values, preferences, and circumstances.

In STS, it might be that there are more reasonable options because of uncertainty around treatment outcomes, a trade-off of risks and benefits and/or patient preferences.

26. In which percentage of eSTS patients do you think that more than one reasonable treatment option exists

- Slider 0–100%

27. Do you discuss the reasonable treatment options with the patient to decide together with the patient about the personalized treatment plan for the patient?

- □ never     □seldom     □ sometimes     □ often     □ always

Below are a few arguments for the use of **RPM** to support (shared) decision-making in eSTS patients. Please select the statement(s) to which you are in agreement? (multiple answers possible)

28. The use of RPM stimulates me to:

- □ jointly decide about treatment with the patient
- □ discuss pros and cons of different treatment options with the patient
- □ involve my patients in decision-making
- □ increase patients’ knowledge about risks and benefits of treatment options
- □ better align treatment decisions with patients’ values and goals

29. With the following statements we want to assess general attitudes of STS professionals towards shared decision-making (SDM). (Please scroll bar)

Is SDM a necessary aspect of clinical practice in sarcoma care? Unnecessary for clinical care (0)—Necessary for clinical care (100)

Is SDM welcomed by STS patients?

Not welcomed by most patients (0)—Welcomed by most patients (100)

Is SDM a good use of STS professional’s time?

Poor use of my time with patients (0)—Good use of my time with patients (100)

Are STS professionals confident using SDM in clinical practice in sarcoma care?

A skill I do not feel confident using in clinical practice (0)—A skill I feel confident using in clinical practice (100)

Is SDM important in situations where there are strong clinical recommendations?

Not important if there is a strong clinical preference (0)—Important even if there is a strong clinical preference (100)”

## Appendix 3 Stratified results of the use of RPMs and perspectives on shared decision-making according to continent and specialty

See Tables

**Table 3:** Use of RPM during tumor board according to continent

	Europe (*n* = 143)	North America (*n* = 76)	Asia (*n* = 39)
Never	31 (21.7)	28 (36.8)	23 (59.0)
Seldom	18 (12.6)	11 (14.5)	8 (20.5)
Sometimes	40 (28.0)	20 (26.3)	7 (17.9)
Often	35 (24.5)	12 (15.8)	1 (2.6)
Always	19 (13.3)	5 (6.6)	–
Missing	–	–	–

3,

**Table 4:** Use of RPM during consultation according to continent

	Europe (*n* = 143)	North America (*n* = 76)	Asia (*n* = 39)
Never	39 (27.3)	23 (30.3)	23 (59.0)
Seldom	21 (14.7)	13 (17.1)	5 (12.8)
Sometimes	38 (26.6)	21 (27.6)	7 (17.9)
Often	28 (19.6)	11 (14.5)	1 (2.6)
Always	7 (4.9)	1 (1.3)	–
Missing	10 (7.0)	7 (9.2)	3 (7.7)

4,

**Table 5:** Use of RPM during tumor board according to specialty

	Orthopedic oncologist (*n* = 127)	Surgical oncologist (*n* = 40)	Medical oncologist (*n* = 79)	Radiation oncologist (*n* = 25)
Never	46 (36.2)	9 (22.5)	16 (20.3)	11 (44.0)
Seldom	29 (22.8)	6 (15.0)	6 (7.6)	1 (4.0)
Sometimes	29 (22.8)	15 (37.5)	22 (27.8)	6 (24.0)
Often	19 (15.0)	7 (17.5)	19 (24.1)	6 (24.0)
Always	4 (3.1)	3 (7.5)	16 (20.3)	1 (4.0)
Missing	–	–	–	–

5,

**Table 6:** Use of RPM during consultation according to specialty

	Orthopedic oncologist (*n* = 127)	Surgical oncologist (*n* = 40)	Medical oncologist (*n* = 79)	Radiation oncologist (*n* = 25)
Never	61 (48.0)	5 (12.2)	12 (15.2)	8 (32.0)
Seldom	26 (20.5)	8 (19.5)	6 (7.6)	3 (12.0)
Sometimes	25 (19.7)	16 (39)	24 (30.4)	6 (24.0)
Often	10 (7.9)	7 (17.1)	22 (27.8)	4 (16.0)
Always	–	1 (2.4)	5 (6.3)	2 (8.0)
Missing	5 (3.9)	4 (9.8)	10(12.7)	2 (8.0)

6,

**Table 7:** In which percentages of patients do you think (shared) decision-making is applicable according to specialty?

	Orthopedic oncologist (*n* = 116)	Surgical oncologist (*n* = 36)	Medical oncologist (*n* = 69)	Radiation oncologist (*n* = 21)
Mean (95% CI)	55 (50–60)	47 (40–55)	55 (50–61)	44 (33–56)

7 and

**Table 8:** In which percentages of patients do you think (shared) decision-making is applicable according to continent?

	Europe (*n* = 127)	North America (*n* = 65)	Asia (*n* = 36)
Mean (95% CI)	50 (46–54)	58 (52–64)	58 (49–67)

8.
